# Web and MATLAB-Based Platform for UAV Flight Management and Multispectral Image Processing

**DOI:** 10.3390/s22114243

**Published:** 2022-06-02

**Authors:** Nourdine Aliane, Carlos Quiterio Gomez Muñoz, Javier Sánchez-Soriano

**Affiliations:** 1 Industrial and Aerospace Engineering Department, Universidad Europea de Madrid, 28670 Villaviciosa de Odón, Madrid, Spain; carlosquiterio.gomez@universidadeuropea.es; 2 Escuela Politécnica Superior, Universidad Francisco de Vitoria, 28223 Pozuelo de Alarcón, Madrid, Spain; javier.sanchez@ufv.es

**Keywords:** unmanned aerial vehicle (UAV), precision agriculture (PA), remote sensing (RS), multispectral image processing, MATLAB^®^, web programming

## Abstract

The deployment of any UAV application in precision agriculture involves the development of several tasks, such as path planning and route optimization, images acquisition, handling emergencies, and mission validation, to cite a few. UAVs applications are also subject to common constraints, such as weather conditions, zonal restrictions, and so forth. The development of such applications requires the advanced software integration of different utilities, and this situation may frighten and dissuade undertaking projects in the field of precision agriculture. This paper proposes the development of a Web and MATLAB-based application that integrates several services in the same environment. The first group of services deals with UAV mission creation and management. It provides several pieces of flight conditions information, such as weather conditions, the KP index, air navigation maps, or aeronautical information services including notices to Airmen (NOTAM). The second group deals with route planning and converts selected field areas on the map to an UAV optimized route, handling sub-routes for long journeys. The third group deals with multispectral image processing and vegetation indexes calculation and visualizations. From a software development point of view, the app integrates several monolithic and independent programs around the MATLAB Runtime package with an automated and transparent data flow. Its main feature consists in designing a plethora of executable MATLAB programs, especially for the route planning and optimization of UAVs, images processing and vegetation indexes calculations, and running them remotely.

## 1. Introduction

The rapid development of unmanned aerial vehicles (UAVs) combined with the growth in the use of information and communication technologies (ICT) have had a great impact in the agriculture field [1]. These emerging technologies have created a new paradigm that offers new perspectives to modify existing procedures and develop innovative applications in precision agriculture (PA), as well as it promises a long-term increase of productivity [2].

Nowadays, UAVs are economically affordable and their ability to be flown at different altitudes and speeds make them helpful for photogrammetry and remote sensing (RS) techniques [3]. Their use is particularly extended in precision agriculture due to their capability to house onboard visible, thermal, and near-infrared sensors for acquiring aerial images of cultivated fields quickly in non-destructive and cost-effective ways [4,5,6,7,8,9,10,11]. In fact, UAVs have been widely used in the last 30 years in precision agriculture [12]. A comprehensive review on UAV-based applications in precision agriculture can be found in [1,4,5,6,7,8,9,10,11,12,13], and several UAV onboard sensors for remote sensing are reviewed in [14]. Deployment of any UAV application involves the integration of several issues. Firstly, such challenge starts with the selection of the sensor to be installed onboard; having in mind that sensor’s parameters may influence the undertaken task. Afterwards, the UAV route planning may require several sub-tasks. For example, it may require path optimization for scanning the field, frequency of images acquisition, and even defining an emergency plan for handling critical situations. Furthermore, the defined route can be validated using some sort of offline simulator. The flight mission is also subject to other issues, such as weather conditions, the geomagnetic deviation (KP index), and other zonal restrictions, assuming that all the low-level issues, such as the motors controllers are properly tuned [15]. Finally, the acquired images may be manually loaded to a host computer for performing their processing.

From the software point of view, deployment of UAV applications requires different sorts of tools and utilities. Beyond the ground control station (GCS) program, the development of the tasks described previously may require the use of specialized software, such as digital-maps, OpenCV, MATLAB, or similar software for image processing, geographical information systems (GIS), programming languages for automating some processes, and even may involve databases. Thus, the development of such applications requires advanced software integration. This situation may frighten and dissuade end-users to undertake projects in the field of precision agriculture.

An example of UAV used in precision agriculture is the known “Parrot Bluegrass” [16], a multipurpose agriculture drone. The Parrot Bluegrass has its own embedded RGB camera and may uses “Parrot Sequoia” [16], a device made up of multiple multispectral sensor, where each capture a different wavelength of light. Images and data are temporally saved within an internal memory, which are then transferred to a computer, where the final processing of the collected data may be achieved using third-party apps or tools like Pix4fields [17] or Airinov [16], a cloud-based platform dedicated to Parrot Bluegrass and provides farmers with services to manage their crops, determine optimum fertilizer application. As UAV flight management is concerned, it is worth to mention Pix4Dfields [17], a suite of independents apps designed to support farmer in their agriculture workflow. This suite provides Pix4DCapture [18], an app for mapping areas with customized parameters like image overlap, camera angle and flight altitude according to user needs, and it implements five types of scanning patterns (polygon, grid, double grid, circular, free flight). Collected standard RGB or multispectral images may be analyzed using tools, such as Pix4DMapper [19], for generating crop health maps based on wavelength info. The main concerns about this suite are about the requirement of the use of the most recent devices and required license subscription. The first concern about the Pix4D suite is that some services, including route planning, are tied to specific drones, namely DJI, Parrot, and Yuneec manufactures, and does not allow the use of other onboard multispectral cameras, and image processing services are carried out using different software tools that are not integrated in the same environment. Another drawback is that the suite does not provide an integrated flight conditions information, such as weather conditions, KP index, air navigation maps, or aeronautical information services, which are key elements for deploying UAVs missions. Finally, the use of this suite requires the latest devices and requires subscription licenses to specific services. This paper proposes the design and development of a Web and MATLAB-based application that integrates several functionalities required in precision agriculture, namely providing services for UAV flight management and services focused on multispectral images processing. From software development point of view, the effort has been made in defining a modular and scalable architecture, organizing several monolithic tools and independent programs in a single application with transparent and automated data flow, allowing a fast and intuitive configuration of end-user services. One of the main features of the proposed application is the integration of several services in the same environment. The first group of services deals with the UAV mission management, allowing the creation of UAV missions and providing several flight conditions information, such as weather conditions, KP index, air navigation maps, or aeronautical information services including notices to Airmen (NOTAM). The second one deals with route planning converting a selected polygonal area on the map to an optimized route that includes sub-route handling for long journeys. Finally, the third group deals with multispectral image processing and vegetation indexes calculation and visualizations. The ultimate goal of this application is to provide farmers and end-users with an easy task-definition ambient and to help them in gaining insights in making their decision.

The rest of the paper is organized as follow: Section 2 describes the main functionalities of the developed Web Application platform from the end-user point of view. Section 3 describes the application software architecture as well as describing the most relevant tools involved its development. Section 4 deals with the web application implementation, where the first part is dedicated to the UAV mission management, and how the information (KP index, weather forecasting and aeronautical information) are aggregated to application. The following subsection is devoted to the route generation explaining the tessellation process for the trajectory waypoints generation, and how flight emergency plan is handled. The third subsection deals with multispectral images processing, putting the focus on the issues related to reflectance correction and vegetation indexes calculation. Section 5 provides some considerations related to simulation and experimental results and provides discussions regarding the validation of the app functionalities. Section 6 ends the paper drawing some conclusions.

## 2. Web Application Functionalities

The developed Web Application provides a broad range of functionalities required in precision agriculture, where the most important are organized in three groups:

*UAV mission management*: The entry point to create missions or retrieve data of saved ones. A mission starts by selecting vertex points of a polygonal area using OpenStreetMap. It allows consulting flight conditions, such as weather conditions, KP index, air navigation maps, and aeronautical information services including notices to Airmen (NOTAM).

*Route planning*: Implements the ray-casting algorithm to convert the selected area on the map to an optimized route that includes sub-route handling for long journeys. Route generation depends on other parameters, such as camera sensors and lens parameters, the ground sample distance (GSD), geometrical shape of the field, etc. Furthermore, generated missions can be exported using MAVLINK exchange format, which is compatible with open programs such as Mission-Planner, to be loaded to a specific UAV platform.

*Multispectral image processing and vegetation indexes calculation*: The developed App provides some functionalities, such as uploading hyperspectral images to the server, performing some pre-processing such as reflectance corrections, calculate several vegetation indices using an interactive viewer, and generate reports as pdf files for their offline consultation.

## 3. Software Architecture

**Figure 1:**
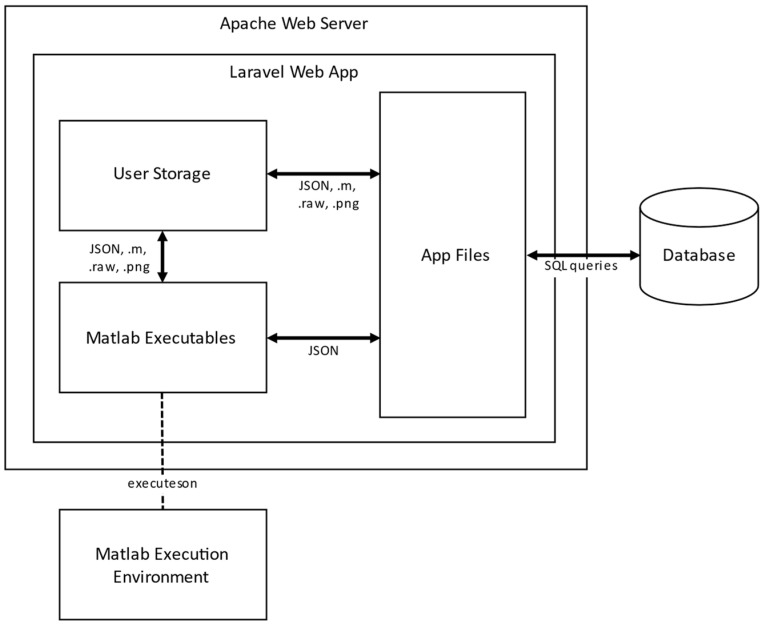
Software architecture and modules organization.

The developed web app relies to a client-server framework, where the provided services run in the back-end (on the server side), and the services are accessed using web browsers. Its main feature is organizing several monolithic tools and independent programs in a single application with transparent and automated data flow, allowing a fast and intuitive configuration. Software architecture in the back-end side is built around four software components: namely Apache-Server, Laravel framework [20] for the Web application deployment, MariaDB database, and MATLAB Runtime and its aggregated executables files. Software modules organization is shown in Figure 1.

The Apache server is used to host the Web application components and the different MATLAB executables modules needed to perform the UAVs route planning, images processing and vegetation indexes calculations. The Web application is built upon Laravel framework using PHP and JavaScript programming languages for handling easily issues related to user accounts, authentication and routing.

**Figure 2:**
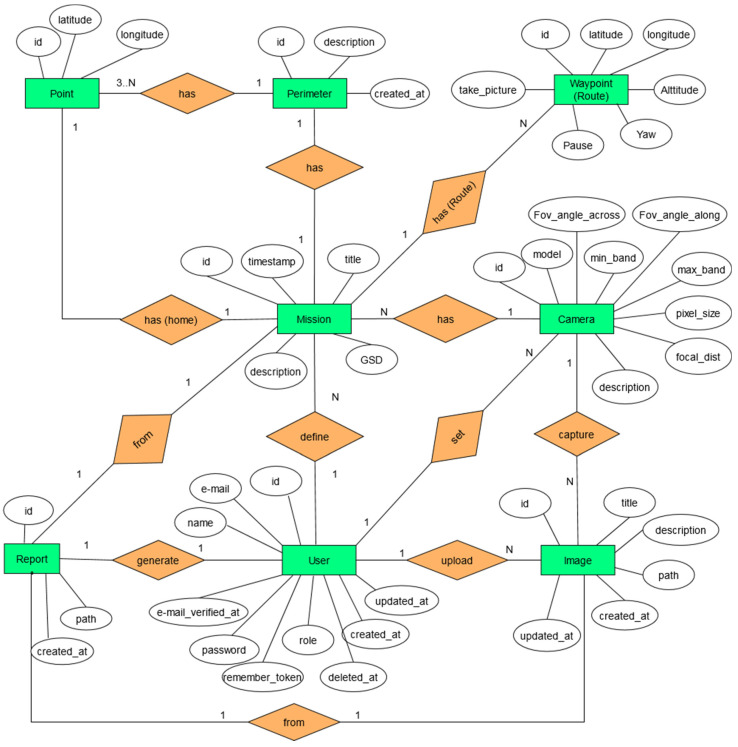
Entity-Relationship diagram of the system database.

The MariaDB database associated with the application consists of ten related tables used for different needs, such as user profiles management, missions definition, cameras parameters, polygon area being studied, waypoints of the associated route, multispectral images management, analysis reports, and so forth. Figure 2 shows the entity-relationship diagram (ERD) of the most important entities. In addition, the database includes a couple of entities for user accounts and passwords recovering. The interactions of end-users as, well as the application itself with the database (queries and data saving), are carried out through a set of pre-programmed scripts in the back-end.

The main feature of the application software architecture is the use MATLAB. The central idea consists in designing a plethora of executables MATLAB programs, generated previously with the MATLAB compiler tool, and run them remotely. The execution of the compiled programs does not need MATLAB environment itself, but only requires the MATLAB Runtime module.

**Figure 3:**
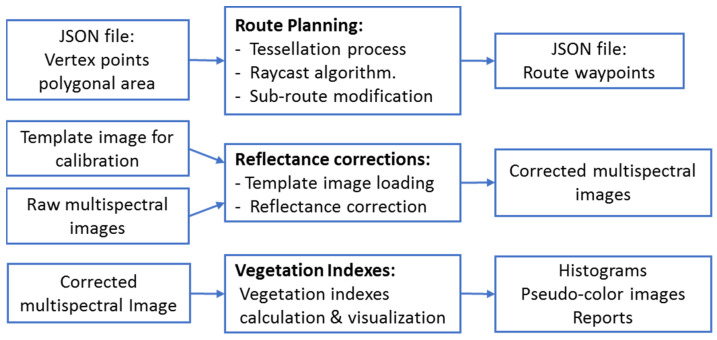
The main MATLAB compiled modules within the application framework.

The most relevant MATLAB compiled programs developed for the application include the route planning module, the reflectance correction, the calculation of 18 vegetation indexes. These binary files are ready to be executed providing the proper input data. In this sense, the execution is automated through scripts allowing a parametrized and fully transparent flow of data. The communication and information exchange between the different modules (web components and MATLAB modules) is handled through JavaScript Object Notation Interchange format (JSON). For example, the call to run a MATLAB module requires, as an input parameter, the path of a JSON file that provides all the required parameters for its execution. In the same way, the outputs after the execution of a MATLAB module (new images, reports, data) are saved, and are referenced by another JSON output file. Therefore, by adhering to the defined communication mechanism, it is possible to integrate in the App with other modules developed in other programming languages. Figure 3 shows the most important compiled MATLAB modules.

Regarding licensing, mention that it is only required a license for MATLAB compiler toolbox during the development phase. Once the executables are generated, tested and validated, they are ready to be installed on the server and do not require any other license for their use.

Finally, user can run several processing concurrently, and processing tasks are handled within independent threads. Finally, the software architecture is scalable, and more processing modules can be added to the server easily by uploading their corresponding compiled MATLAB file.

## 4. Web Application Functionalities Deployment

This section presents the Web application functionalities and provides some details related to their implementation.

### 4.1. UAV Mission Management

Mission management is the main entry point to create new missions or retrieve recorded ones. It allows defining new missions using recorded ones as templates by modifying few parameters. The creation of new mission starts by selecting vertex points of a polygonal area using OpenStreetMap, integrated within the Web application. Adding, deleting, or moving vertex points can modify the polygonal area dynamically, and the selected area may be given features, such as a title, a breve description, and so forth.

**Figure 4:**
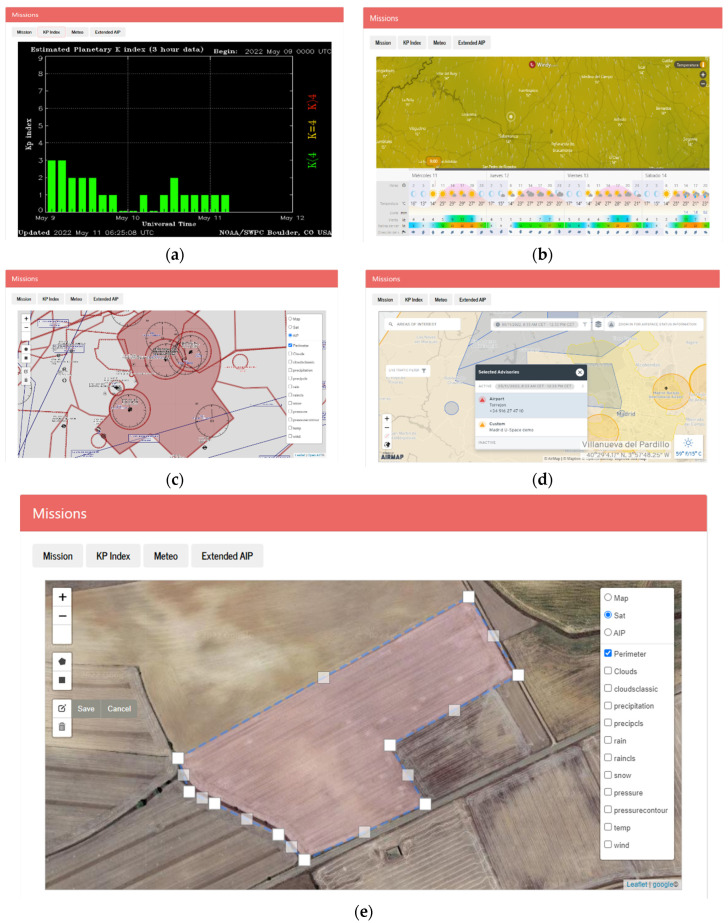
Some snapshots: (**a**) Observation of the KP index, (**b**) Temperature prediction, (**c**) map with AIP information, (**d**) Advanced AIRMAP viewer, (**e**) Route planning.

Furthermore, to provide a comprehensive environment for mission preparation, complementary information, such as KP index, weather forecasting and aeronautical information services including notices to Airmen (or NOTAM) are also visualized. The KP Index is obtained in real time from the national oceanic and atmospheric administration (NOAA) repository [21]. The current meteorological information and its forecast are superposed to the maps and are obtained from the Windy service [22]. Finally, the App displays basic aeronautical information publications (AIP), gathered from OpenAIP service [23], a free web-based aeronautical information platform allowing users to add navigational information. Finally, the App displays through an extended viewer advanced information, such as Notice to Airmen (NOTAM) obtained from the Airmap service [24]. Map layers, weather forecasting, and aeronautical information publication are visualized using OpenStreetMap [25], and their manipulation is handled using Leaflet API and the Draw and Fullscreen plugins [26,27,28]. Figure 4 shows some snapshots of different views for managing with the UAV missions.

### 4.2. Route Planning

**Figure 5:**
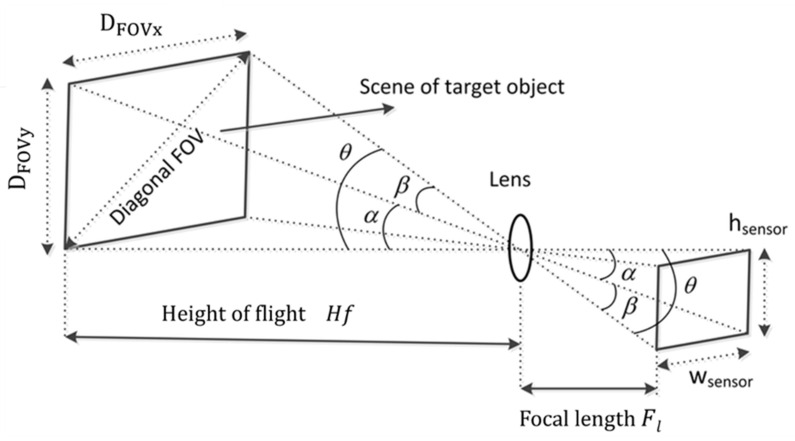
Field of view and camera parameterization.

The route planning consists in generating the UAV flight altitude and a set of waypoints, using entered mission data; namely vertexes of the selected polygonal area, the parameters of the onboard camera as well as the desired ground sampling distance (GSD). GSD defines the distance between two consecutive pixels measured on the ground; a bigger GSD corresponds to lower spatial resolution and with less visible details. The flight height *H_f_* at which the UAV will be flying is calculated [22] according to,

(1) $$H_{f}=\mathrm{GSD}\cdot \frac{F_{l}}{P_{s}}$$

where GSD is the desired ground sampling distance, *F_l_* is the focal distance of the camera lens, and *P_s_* the size of the camera sensor given in pixels. Furthermore, the calculated height combined with the camera parameters permits the calculation of the camera field of view projection, namely the distances on the ground. Thus, an image projected along the camera field of view on the ground, as illustrated in Figure 5, and the projected distances *D_FOVx_* and *D_FOVy_* along the 2-axis frame are calculated as

(2) $$\;D_{FOVx}=2\cdot H_{f}\cdot tang\frac{\beta }{2};\;D_{FOVy}=2\cdot H_{f}\cdot tang\frac{\alpha }{2}$$

where *α* and *β* are vertical and horizontal angles formed between the height and width of the sensor with the focal point respectively.

The projected areas are taken as tiles (or small parts of a mosaic) within a tessellation process, as will be explained later in this section. On the other hand, as the polygon vertices points captured on the map are given as GPS coordinates and subsequent calculation require their conversion to distances in meters. This conversion is performed using the Haversine formula, where the great circle distance d between two GPS locations, given in terms of latitude and longitude: (*φ*_1_, *λ*_1_) and (*φ*_2_, *λ*_2_), is given as:

(3) $$d=2\cdot R\cdot \sin^{-1}\left(\sqrt{\sin^{2}\left(\frac{\varphi_{2}-\varphi_{1}}{2}\right)+\cos\varphi_{1}\cdot \cos\varphi_{2}\cdot \sin^{2}\left(\frac{\lambda_{2}-\lambda_{1}}{2}\right)}\right)$$

where *d* is given in meters, (*φ_i_, λ_i_*) are given in radians and *R* is the earth radius.

#### 4.2.1. Tessellation Process

**Figure 6:**
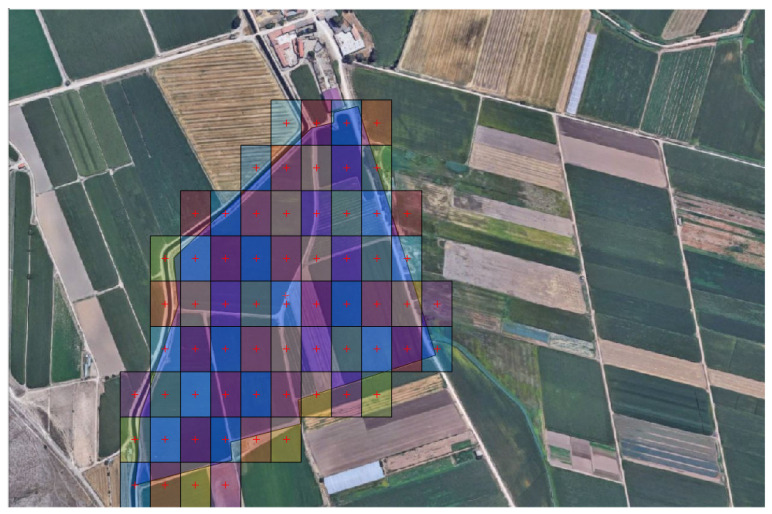
Waypoints obtained from a tessellation process without overlap between tiles. Route starts from the bottom to the top and from the left to the right.

The tessellation process consists in filling the polygon area with tiles, starting from the bottom to the top, and from the left to the right of the selected area, and a tile is kept if it lies inside the polygon. The process for checking whether a tile centre point is within the polygon or not is achieved using the Ray-Casting algorithm [29,30]. The algorithm is implemented in MATLAB through the Raycast function, which indicates if an imaginary ray is thrown out of the point, the number of intersections with the polygon will determine whether this point is within the polygon or not. If the point does not lie within the polygon, the ray will intersect its edge an even number of times. However, if the point lies within the polygon, then it will intersect the edge an odd number of times [31]. Obviously, there are special cases, where tiles center point do not lie inside the polygon, but with one of their vertices lies inside the polygon. These kinds of tiles are discarded, since they lead to an incomplete polygon tessellation. Thus, the algorithm is modified so that tiles with at least one of their vertices are within the polygon are maintained. Figure 6 shows an example of tessellation of an area with no overlap between images.

The previous process does not take into account the overlap between tiles. For a correct union between adjacent tiles, percentages (*Ox*, *Oy*) along the (*D_FOVx_*, *D_FOVy_*) are specified for tiles overlap. Thus, the displacements (*Dx*, *Dy*) between two overlapped tiles are calculated according to:

(4) $$\;D_{x}=D_{FOVx}\cdot O_{x};\;\;D_{y}=D_{FOVy}\cdot O_{y}$$

The displacements (*Dx*, *Dy*) represent the center location of the new tile along the (x-y) axis with respect to the previous one. These displacements are converted into their corresponding GPS coordinates. The displacement along x-axis modifies the longitude and displacement along y-axis modifies the latitude with respect to the previous point. Furthermore, for small displacements, the geodesic distance between two close GPS locations can approximated by its straight-line distance. Therefore, the increments of the latitude Δ*λ* and the longitude Δ*φ* are calculated using the following approximation:

(5) $$\;\Delta \lambda =\frac{180}{\pi R}D_{y},\;\Delta \varphi =\frac{180}{\pi R}D_{x}\;$$

where *R* is the earth radius, the displacements (*Dx*, *Dy*) are given in meters, and the increments in latitude and longitude angles (Δ*λ*, Δ*φ*) are given in degrees.

The conversion process is accumulative and starts from a reference point that corresponds to the left and bottom corner of the polygon, and the remaining waypoints are crossed in an orthogonal zigzag (Up, Right, Down, Right, etc.) until last waypoint is reached. The route plan is, finally, defined and exported as JSON file with four camps: (GPS location, altitude, waiting time, UAV orientation).

#### 4.2.2. Sub-Route Generation and Emergency Plan

**Figure 7:**
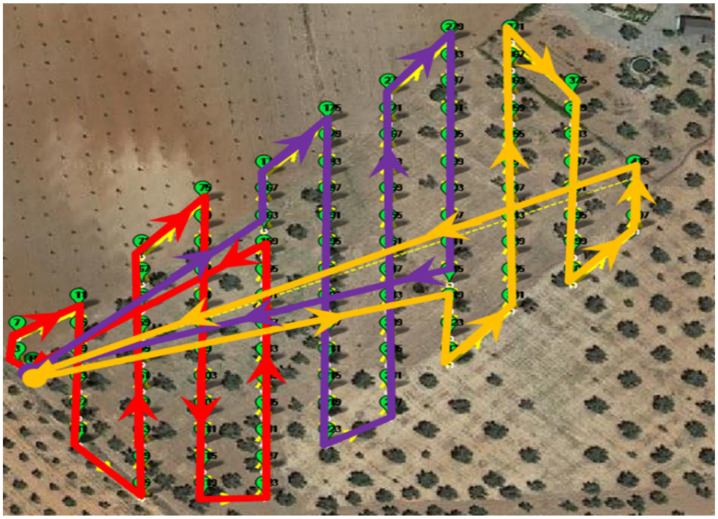
Illustration of a route division into three sub-routes.

Autonomy and batteries charge limitation is a crucial issue when flying UAV, and if the area to be inspected is large enough, batteries my run out before completing the entire mission. In general, common UAV autonomy is about 20 to 30 min depending on the payload and flight conditions. To handle this issue, a generated route is divided into several sub-routes by inserting go-home waypoints. These go-home waypoints are inserted in the route according to a risk evaluation ensuring the UAV to travel from its current position to the next waypoint as well as to be able to return in straight line safely to the go-home position. This risk is evaluated at each waypoint by performing the sum the two distances, and if the resulting distance exceeds a predefined value, a go-home waypoint is then inserted. Figure 7 illustrate a route divided in three sub-routes.

### 4.3. Multispectral Images Processing and Vegetation Indexes Calculation

MATLAB is known as a suitable tool for visual and images processing. However, spectral images present several technical issues, such as reflectance correction due to the atmosphere, managing with their huge size, the implementation from MATLAB point of view of the different indices, and the speeding of the processing using compiled scripts and functions. Thus, this section is dedicated to present some hints and technical solutions to handling these issues.

#### 4.3.1. Reflectance Correction and Calibration

The measured spectral signal is subject to several alterations such as atmospheric effects or surface features. To extract qualitative information from raw signal, image processing and corrections are necessary. It is worth mentioning that, from UAV based hyperspectral imagery point of view, and unlike hyperspectral satellite imagery, the atmospheric alteration is negligible. However, the radiance reflected from the surface requires specific processing, known as surface reflectance correction. Reflectance is a property of the field surface and is independent of incident radiation. The correction of the measurement values using the surface reflectance factors improves signal consistency and data quality.

**Figure 8:**
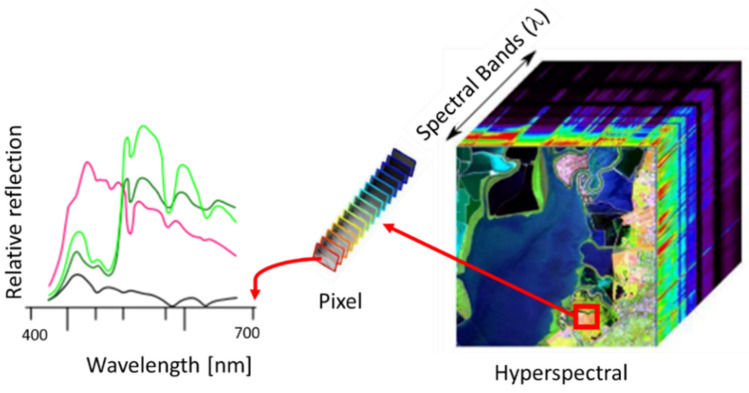
Example of a hyperspectral image, where each pixel consists of a complete reflection spectrum at its position.

In spectroscopy, calibration techniques and surface reflectance correction, in general, use linear regression models, which is mainly a linear relation between the reflectance correction factor versus the wavelength variables. The calibration process is subject to two mathematical issues: handling several wavelengths and their correlation [32]. Figure 8 shows an example of hyperspectral image where the reflectance of the same spatial position (or a pixel) is shown along the different wavelengths.

**Figure 9:**
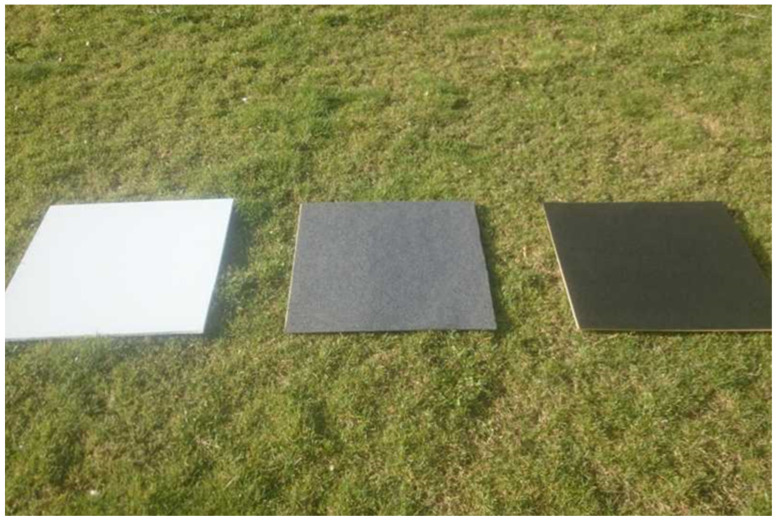
Templates for reflectance calibration, (Image from MosaicMill).

In this sense, calibration process is performed using a template with several known emissivities that includes a pure black, white and some intermediates gray colors [33,34]. An example of such template taken from MosaicMill [35] is shown in Figure 9.

**Figure 10:**
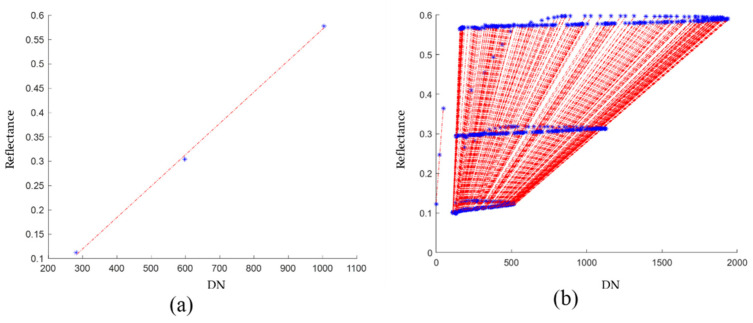
Emissivities versus wavelength: (**a**) linear regression for each pixel of different emissivities at a specific wavelength. (**b**) Linear regression for each pixel of different emissivities for all the acquired wavelengths. The asterisks correspond to the emissivity percentage of the colors of the template for different wavelengths: Black (10%), Gray (30%) and White (57%).

In this process, a 3D matrix of the emissivities of the template at each wavelength, as well as the average emissivity for template tonalities, were obtained [36,37]. It is also obtained the relationship between the camera digital number *DN_λ_* and the reflectance percentage of each template tonalities (being 0% for the black and 100% for the white color). Finally, the parameters of the linear regression that approximates these points for each of the wavelengths in the image are obtained. (See Figure 10).

**Figure 11:**
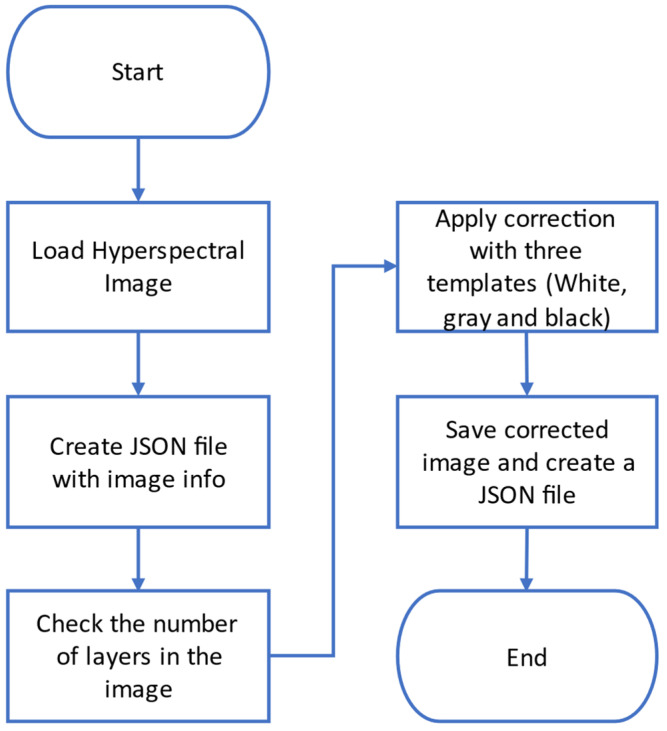
Flowchart followed in MATLAB for multispectral images correction.

From MATLAB point of view, this correction consists in a multiplication of each pixel (in the matrix at each spectral band) with its corresponding correction values. A new cubic hyperspectral image is obtained by applying the correction to all the layers. Figure 11 shows the flowchart of the algorithm used to generate the corrected hyperspectral image.

#### 4.3.2. Vegetation Indexes Calculation and Visualization

After reflectance correction, images are ready to be processed, which consists mainly in extracting information from specific hyperspectral images layers to calculate the different vegetation indexes. Most of these indices are based on the interactions between vegetation and electromagnetic radiation in the red and infrared spectrum bands. Vegetation indexes are quantitative measurement to estimate plants vigor and the vegetation status and health, and they are expressed as combinations or ratios of the bands of the hyperspectral images, allowing distinguishing reflectance in different areas in the examined filed. In this sense, a number of vegetation indices have been developed aimed at helping vegetation and filed monitoring.

From MATLAB implementation point of view, these indices are calculated by means of point-by-point operation with two or more reflectance bands and their calculations are usually given as a ratio. To illustrate the main steps involved in the indexes calculations, let us take the NDVI (Normalized Difference Vegetation Index), which is one of the most used in remote vegetation sensing, where the index is calculated using the formula:

(6) $$\mathrm{NDVI}\;=\frac{\lambda_{IR}-\lambda_{RED}}{\lambda_{IR}+\lambda_{RED}}$$

where *λ_IR_* is the infrared band, specifically it corresponds to the near infrared, with a wavelength closer to 860 nm; *λ_RED_* corresponds to the band of the red color visible spectrum with a wavelength close to 640 nm.

**Figure 12:**
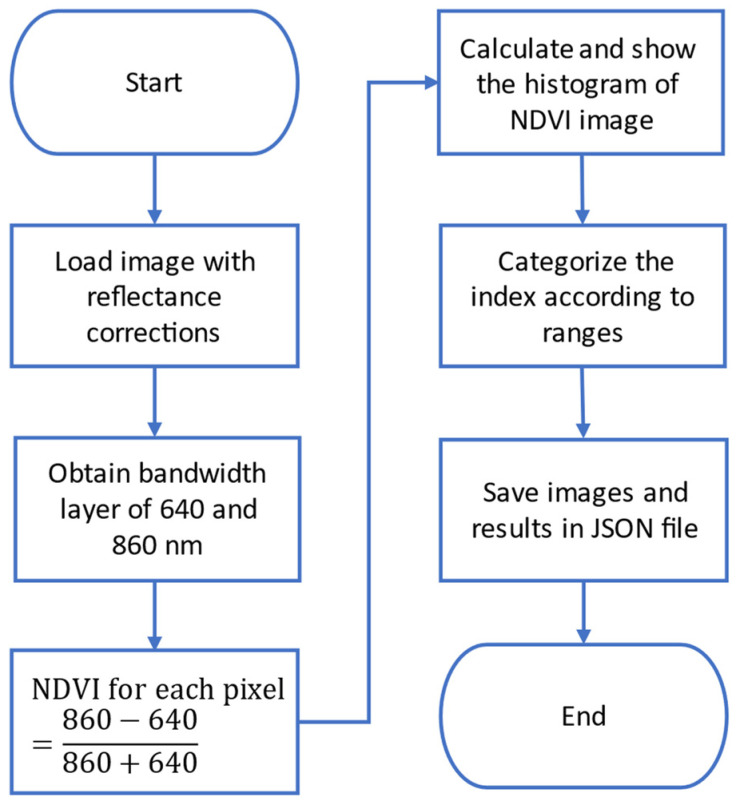
Flowchart of the main steps in the NDVI index processing.

**Figure 13:**
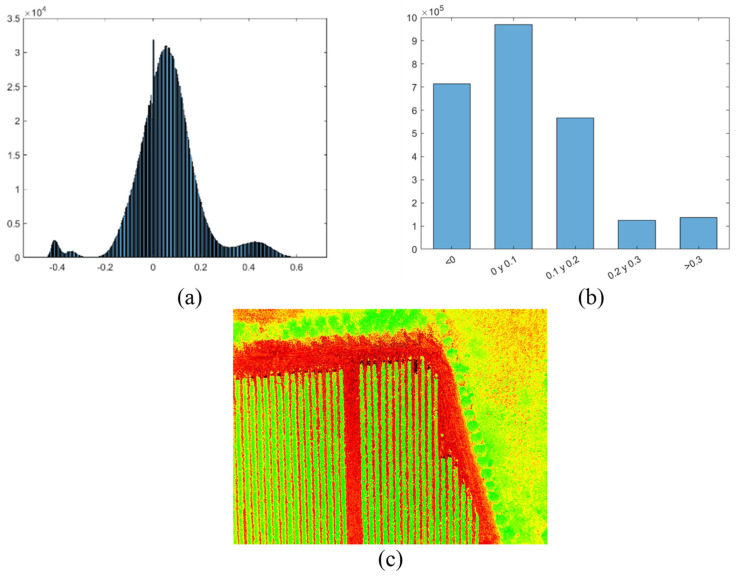
Snapshots of NDVI visualization: (**a**) Histogram. (**b**) Categorized histogram. (**c**) Pseudo-color image.

The resulting calculation is a new matrix, where each element corresponds to the value of the index calculated at the corresponding pixel. Afterwards, this resulting matrix may be used in more calculation. For example, visualization of the histogram of the NDVI matrix displays the shape of the underlying distribution and shows clearly the most predominant indices organized in bins. Another processing deals with classification and categorization of the values according to a given ranges of interest of the index. For the NDVI case, the values are classified whether or not the values are greater than 0.3, which corresponds to healthy state of the vegetation. Finally, the NDVI matrix is saved as a pseudo-color image. Figure 12 shows the flowchart for the main steps followed in the NDVI index processing, and Figure 13 shows some snapshots of its corresponding visualization.

The developed Web application is not limited to the calculation of the NDVI index, but it includes the implementation of the following indexes:

NDVI: Normalized Difference Vegetation Index
RVI: Ratio Vegetation Index
EVI: Enhanced Vegetation Index
SSC: Soluble Solid Content
SAVI: Soil Adjusted Vegetation Index
CCI: Citrus Color Index
ARVI: Atmosphere Resistant Vegetation Index
GCI: Green Chlorophyll Index
MCARI: Modified Chlorophyll Absorption in Reflectance Index
DCNI: Double-peak Canopy Nitrogen Index
SIPI: Structure Insensitive Pigmentation Index
NBR: Normalized Calcination Index
DNBR: Difference Normalized Calcination Index
NDWI: Normalized Difference Water Index
PLS: Regression Water Stress Index
PRI: Photochemical Reflectance Index
PSRI: Plant Senescence Reflectance Index
TA: Titratable Acidity

**Figure 14:**
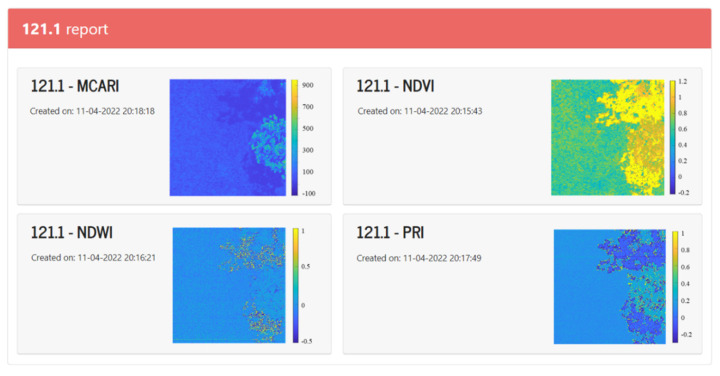
Snapshot of (MCARI, NDVI, NDWI, and PRI) indexes visualization.

The indexes matrices are visualized as pseudo-color images, where each pixel corresponds to the value of the calculated index at the corresponding location in the hyperspectral image. This visualization is achieved automatically with specific MATLAB functions. Figure 14 shows an example of a visualization of four indexes.

## 5. Simulation and Experimental Results

**Figure 15:**
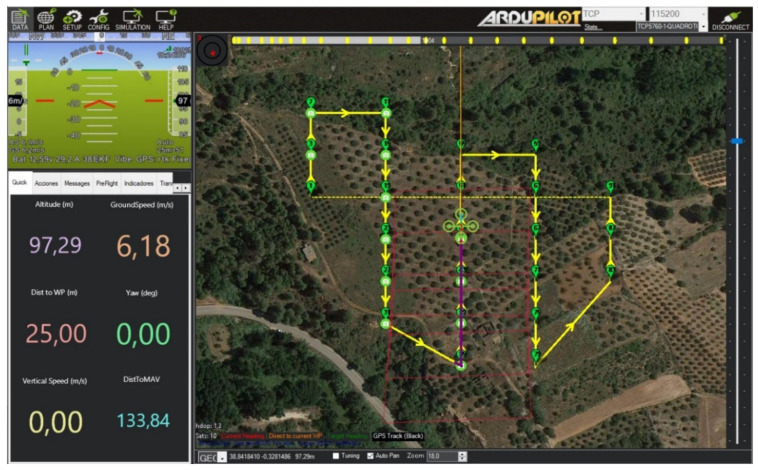
Mission-Planner and SITL environment.

The route planning is assessed in a simulation as well as in real fly. For simulation purpose, the route planning is assessed in a simulation environment using the ArduPilot software tools [38]. Firstly, a mission is generated and then downloaded from the Web application as a plain file. Afterwards, the Mission-Planner program [39], a ground control station for UAV control, is used to simulate UAV flights using its built-in simulation in the loop (SITL) program [40]. The routes planning assessment has been performed carrying out several simulations, contrasting visually that the UAV follows the waypoints of the planned routes with the desired orientation. Figure 15 shows a snapshot of the SITL environment illustrating a route followed by a 4-rotor UAV.

**Figure 16:**
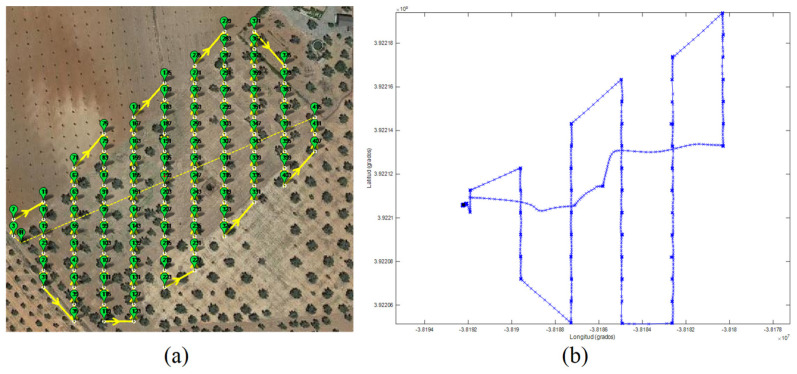
(**a**) A mission visualized in Mission-Planner program. (**b**) The actual sub-route recording UAV real GPS waypoints.

**Figure 17:**
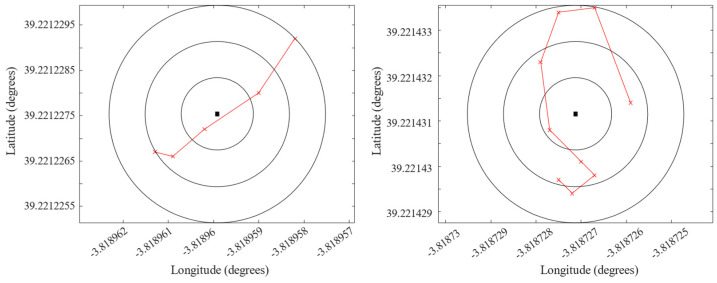
Error position analysis for two random waypoints.

Simulations are also contrasted with real flights to confirm the correctness and the completeness of the generated missions. The same set of waypoints used in a simulation may be loaded in a real UAV. The tests are driven using a DJI S900 Drone with an onboard RTK system. The flights have been set at a height of 30 m, and the drone is programed to stay still 5 s at each waypoint for taking images. The mission contains 418 commands, where 105 are related to go-waypoint commands, and the mission includes a go-home command for seeing the drone behavior against sub-route programming. The flights are supervised using mission-planner program. The route deployment is illustrated in Figure 16a, showing the go-home command at the end of the sub-route. Actual drone positions are tracked using an onboard RTK system. Figure 16b shows the real drone GPS tracking in the field during its first sub-route. The sub-route is scheduled to last 12 min before returning to the home position for batteries change. Figure 17 shows the deviation of the drone trajectory obtained the onboard RTK telemetry, with respect to two random waypoints representing the common center of concentric circles with a ring of 10 cm.

As far as the application execution is concerned, the route generation and the interaction with the backend or interfaces do not have a remarkable need in terms of RAM memory. However, multispectral images processing with MATLAB is demanding. In this sense, the MATLAB runtime itself requires almost 1.5 GB, and it worth it to mention that multispectral image processing requires almost twice as much memory as the size of the images themselves. For example, MATLAB routine requires almost 3.0 GB for processing an image of 1.3 GB. Thus, the designed Web application requires about 8 GB of RAM memory, and if the images to be processed grow in size, memory requirement would grow according to the aforementioned relationship with the size of the multispectral images. The application is tested on a computer with 16 GB RAM showing a fluent and satisfactory interaction.

Finally, the developed application has been tested following a comprehensive plan with more than 300 tests, which are categorized in Table 1. Unit testing were carried out to verify the proper operation of the different modules separately, namely the frontend, backend, and MATLAB modules services. The tests were automated and the results were contrasted with visual inspections to ensure the compliance with the expected results. Some of these tests include the verifications and the validation of the entries into user forms, data saving and retrieving from the database, and MATLAB modules were also tested separately to ensure the correct invocation and execution of the executable modules and scripts files. Integration testing was also performed to verify the proper flow of data between the frontend and backend, and the proper communications between the web components and MATLAB modules through specific JSON files. Systems testing in terms of memory usage or response times, as well as end-user acceptance tests, have been performed. Finally, the App is designed with a responsive web interface, and therefore, usability and access requirements are tested using different devices (smartphone, tablet, PC) with different screen sizes.

## 6. Conclusions

In this paper is presented the development of a Web application for precision agriculture, which combines classical web programming tools and MATLAB Runtime package. The main idea consists in designing a plethora of executables MATLAB programs, especially for UAVs route planning and optimization, images processing and vegetation indexes calculations, and run them remotely. The execution of the MATLAB executable files is automated through a number of scripts, where the information exchange is handled through JavaScript Object Notation Interchange (JSON) format, allowing transparent flow of data. The main advantage of this approach is the achievement of a robust and reliable application, since MATLAB is a well-known and proven tool in scientific fields, especially in image processing. Finally, the software architecture is scalable, and more processing modules can be added to the server easily by uploading their corresponding compiled MATLAB file.

The developed application has been tested by generating several missions, which have been then executed using Mission-Planner and its SITL program, and by contrasting the drone flying over the selected area superposed to the map according to the scheduled flight plan. Several tests are also carried out using a DJI S900 Drone. In the same way, the image processing and vegetal indexes calculation have been tested running the corresponding programs remotely and contrasting the results with expected ones.

The present development presents a number of open questions for future development. Firstly, thanks to the modular and scalable App architecture, it is possible the upgrade the application with a new service to automate the uploading of images to the server and automating the template detection as well as reflectance correction. Another consideration is related to integrate predictive models based on the historical indices obtained for a given field to predict the evolution of the crop. Finally, it is also under consideration how to include within Web and MATLAB-based framework the “MATLAB UAV toolbox” for creating an optimal ecosystem for UAV fleets management, where some ideas are already undertaken in [41].

## Figures and Tables

**Table 1:** Types of testing carried out on the web application.

Test	Visual Inspection	Automated Test	Total
Backend unit tests	23	46	69
Frontend unit tests	20	40	60
MATLAB unit tests	18	36	54
Integration		34	34
System		15	15
Acceptance		80	80
Total	61	251	312

## References

[1] Radoglou-Grammatikis P., Sarigiannidis P., Lagkas T., Moscholios I. (2020). A compilation of UAV applications for precision agriculture. Comput. Netw..

[2] Mylonas P., Voutos Y., Sofou A. (2019). A Collaborative Pilot Platform for Data Annotation and Enrichment in Viticulture. Information.

[3] Colomina I., Molina P. (2014). Unmanned aerial systems for photogrammetry and remote sensing: A review. ISPRS J. Photogramm. Remote Sens..

[4] Tsouros D.C., Bibi S., Sarigiannidis P.G. (2019). A Review on UAV-Based Applications for Precision Agriculture. Information.

[5] Matese A., Toscano P., Di Gennaro S.F., Genesio L., Vaccari F.P., Primicerio J., Belli C., Zaldei A., Bianconi R., Gioli B. (2015). Intercomparison of UAV, Aircraft and Satellite Remote Sensing Platforms for Precision Viticulture. Remote Sens..

[6] Poblete T., Ortega-Farías S., Moreno M.A., Bardeen M. (2017). Artificial Neural Network to Predict Vine Water Status Spatial Variability Using Multispectral Information Obtained from an Unmanned Aerial Vehicle (UAV). Sensors.

[7] Barbedo J.G.A., Koenigkan L.V., Santos T.T., Santos P.M. (2019). A Study on the Detection of Cattle in UAV Images Using Deep Learning. Sensors.

[8] Mazzia V., Comba L., Khaliq A., Chiaberge M., Gay P. (2020). UAV and Machine Learning Based Refinement of a Satellite-Driven Vegetation Index for Precision Agriculture. Sensors.

[9] Niu H., Hollenbeck D., Zhao T., Wang D., Chen Y. (2020). Evapotranspiration Estimation with Small UAVs in Precision Agriculture. Sensors.

[10] Zhang C., Valente J., Kooistra L., Guo L., Wang W. (2021). Orchard management with small unmanned aerial vehicles: A survey of sensing and analysis approaches. Precis. Agric..

[11] Kasimati A., Espejo-García B., Darra N., Fountas S. (2022). Predicting Grape Sugar Content under Quality Attributes Using Normalized Difference Vegetation Index Data and Automated Machine Learning. Sensors.

[12] Mulla D.J. (2013). Twenty five years of remote sensing in precision agriculture: Key advances and remaining knowledge gaps. Biosyst. Eng..

[13] Del Cerro J., Cruz Ulloa C., Barrientos A., de León Rivas J. (2021). Unmanned Aerial Vehicles in Agriculture: A Survey. Agronomy.

[14] Mesas-Carrascosa F.J. (2020). UAS-Remote Sensing Methods for Mapping, Monitoring and Modeling Crops. Remote Sens..

[15] Castillo-Zamora J., Camarillo-Gómez K., Pérez-Soto G., Rodriguez J. (2018). Comparison of PD, PID and Sliding-Mode Position Controllers for V–Tail Quadcopter Stability. IEEE Access.

[16] Parrot-Bluegrass. https://www.parrot.com/assets/s3fs-public/2021-09/bd_bluegrass_productsheet_en_210x297_2018-03-01.pdf.

[17] Pix4Dfields. https://www.pix4d.com/product/pix4dfields.

[18] Pix4DCapture. https://www.pix4d.com/product/pix4dcapture.

[19] Pix4DMapper. https://www.pix4d.com/product/pix4dmapper-photogrammetry-software.

[20] LARAVEL. https://laravel.com/docs/8.x.

[21] NOAA. http://www.n3kl.org/.

[22] WINDY. https://www.windy.com.

[23] OpenAIP. http://maps.openaip.net/.

[24] AIRMAP. https://www.airmap.com.

[25] Open-Street-Map. https://www.openstreetmap.org.

[26] Leaflet-API. https://leafletjs.com/reference-1.7.1.html.

[27] Draw. https://github.com/Leaflet/Leaflet.draw.

[28] Full-Screen. https://github.com/Leaflet/Leaflet.fullscreen.

[29] Gómez Muñoz C.Q., Paredes Alvarez C., Garcia Marquez F.P. (2020). Smart Farming: Intelligent Management Approach for Crop Inspection and Evaluation Employing Unmanned Aerial Vehicles. Proceedings of the International Conference on Management Science and Engineering Management.

[30] Ye Y., Guangrui F., Shiqi O. (2013). An Algorithm for Judging Points Inside or Outside a Polygon. Proceedings of the 2013 Seventh International Conference on Image and Graphics.

[31] Petershofen M. MATLAB Central File Exchange. https://www.mathworks.com/matlabcentral/fileexchange/62227-raycasting.

[32] Polder G., Pekkeriet E.J., Snikkers M. A Spectral Imaging System for Detection of Botrytis in Greenhouses. Proceedings of the EFITA-WCCA-CIGR Conference “Sustainable Agriculture through ICT Innovation”.

[33] Cao S., Danielson B., Clare S., Koenig S., Campos-Vargas C., Sanchez-Azofeifa A. (2019). Radiometric calibration assessments for UAS-borne multispectral cameras: Laboratory and field protocols. ISPRS J. Photogramm. Remote Sens..

[34] Poncet A.M., Knappenberger T., Brodbeck C., Fogle M., Shaw J.N., Ortiz B.V. (2019). Multispectral UAS Data Accuracy for Different Radiometric Calibration Methods. Remote Sens..

[35] MosaicMill. https://www.mosaicmill.com/products_other/reflectance_targets.html.

[36] Zarzar C.M., Dash P., Dyer J.L., Moorhead R., Hathcock L. (2020). Development of a Simplified Radiometric Calibration Framework for Water-Based and Rapid Deployment Unmanned Aerial System (UAS) Operations. Drones.

[37] Ortiz J.D., Avouris D., Schiller S., Luvall J.C., Lekki J.D., Tokars R.P., Becker R. (2017). Intercomparison of approaches to the empirical line method for vicarious hyperspectral reflectance calibration. Front. Mar. Sci..

[38] ARDUPILOT. https://ardupilot.org/.

[39] MISSION-Planner. https://ardupilot.org/planner/.

[40] SITL. https://ardupilot.org/dev/docs/sitl-simulator-software-in-the-loop.html.

[41] Bemposta Rosende S., Sánchez-Soriano J., Gómez Muñoz C.Q., Fernández Andrés J. (2020). Remote Management Architecture of UAV Fleets for Maintenance, Surveillance, and Security Tasks in Solar Power Plants. Energies.

